# Consent Codes: Upholding Standard Data Use Conditions

**DOI:** 10.1371/journal.pgen.1005772

**Published:** 2016-01-21

**Authors:** Stephanie O. M. Dyke, Anthony A. Philippakis, Jordi Rambla De Argila, Dina N. Paltoo, Erin S. Luetkemeier, Bartha M. Knoppers, Anthony J. Brookes, J. Dylan Spalding, Mark Thompson, Marco Roos, Kym M. Boycott, Michael Brudno, Matthew Hurles, Heidi L. Rehm, Andreas Matern, Marc Fiume, Stephen T. Sherry

**Affiliations:** 1 Centre of Genomics and Policy, Faculty of Medicine, McGill University, Montreal, Quebec, Canada; 2 Broad Institute of MIT and Harvard, Cambridge, Massachusetts, United States of America; 3 Centre for Genomic Regulation (CRG), Barcelona, Spain; 4 Universitat Pompeu Fabra (UPF), Barcelona, Spain; 5 Office of Science Policy, Office of the Director, National Institutes of Health, Bethesda, Maryland, United States of America; 6 Department of Genetics, University of Leicester, Leicester, United Kingdom; 7 European Molecular Biology Laboratory—European Bioinformatics Institute (EMBL—EBI), Wellcome Trust Genome Campus, Hinxton, Cambridge, United Kingdom; 8 Human Genetics Department, Leiden University Medical Center, Leiden, The Netherlands; 9 Children’s Hospital of Eastern Ontario Research Institute, University of Ottawa, Ottawa, Ontario, Canada; 10 Centre for Computational Medicine, Hospital for Sick Children, Toronto, Ontario, Canada; 11 Department of Computer Science, University of Toronto, Toronto, Ontario, Canada; 12 Wellcome Trust Sanger Institute, Wellcome Genome Campus, Hinxton, Cambridge, United Kingdom; 13 Department of Pathology, Brigham & Women's Hospital and Harvard Medical School, Boston, Massachusetts, United States of America; 14 Bioreference Laboratories, Inc., Elmwood Park, New Jersey, United States of America; 15 DNAstack, Toronto, Ontario, Canada; 16 National Centre for Biotechnology Information, US National Library of Medicine, Bethesda, Maryland, United States of America; Stanford University School of Medicine, UNITED STATES

## Abstract

A systematic way of recording data use conditions that are based on consent permissions as found in the datasets of the main public genome archives (NCBI dbGaP and EMBL-EBI/CRG EGA).

## Overview

Restrictions on data use may be necessary in order to respect the consent of research participants and patients. As members of the Global Alliance for Genomics and Health (Global Alliance), we examined the variation in data use conditions that are based on consent provisions for genomics datasets in research and clinical settings based on the following: (1) a review of guidance from the National Institutes of Health; (2) a survey of data use conditions for data accessed within a large research institute, the Broad Institute of MIT and Harvard, and for data held within the European Genome-phenome Archive (EGA) of the European Bioinformatics Institute (EMBL-EBI) and Centre for Genomic Regulation (CRG); and (3) consultation with the international scientific community via the Global Alliance Data Working Group and with the Matchmaker Exchange Project. Based on our study, we propose a structure for recording data use “categories” and “requirements” with a view to support maximum data use and integration.

## Introduction

The Global Alliance for Genomics and Health (Global Alliance; [1]) is an international coalition dedicated to increasing the potential of genomic medicine to advance human health through effective and responsible data sharing. To this end, it has established the Framework for Responsible Sharing of Genomic and Health-Related Data [2]. In addition, the Beacon Project is a key Global Alliance project launched as a test of the willingness of international research and clinical sites to share genomic data in the simplest of all technical contexts. Its goal is to address barriers to international genomic data sharing by fostering the creation and development of “Beacons” that share data in a manner that respects the interests of research participants. A Beacon is a simple public web service that any institution can implement, designed to accept simple queries such as "Do you have record of any genomes with an 'A' at position 100,735 on chromosome 3?" [3].

From an ethical, legal, and social perspective, the Beacon Project aims to resolve core data sharing issues: how to enable broad data access and translation while protecting and promoting the interests and rights of research participants and patients. Respecting the terms of the consent of individuals presents challenges as large-scale datasets are shared and analyzed in numerous research studies. Expertise in interpreting permissible uses of data based on the original informed consent process represents a significant research cost in terms of both time delays and financial expense. These could be reduced if conditions of data use and sharing were clearly communicated and recorded at the time of data generation. This would also facilitate respecting the appropriate secondary research use of data. Here, we examine the variation in data use conditions that are based on consent provisions for genomics datasets in both the research and clinical settings. A large proportion of these datasets are shared through data access mechanisms, which allow for limitations to research uses of the data according to their compatibility with research participant and patient consent (“registered,” “controlled,” or “managed” access). Based on our study of existing data use restrictions, we propose a structure for recording data use “categories” and “requirements” with a view to supporting maximum data use and integration, as well as the inclusion of consent information within projects like the Beacon Project.

## Results and Discussion

The proposed data use categories and requirements (“‘Consent Codes”) to be used to assign genomic datasets to standardized data use groups so as to facilitate their reuse (i.e., consistent interpretation for appropriate secondary data uses and the ability for integration with other data) are shown in Table 1. It is important to note that this proposal derives from our knowledge of current data use conditions based on past analysis of various research participant and patient consent protocols, many of which are for legacy data collections that preceded the era of both big data and broad data sharing policy (for guidance on legacy consents and international data sharing, see the Global Alliance consent tools [4]). In particular, it is not intended as a suggestion of how consent ought to be sought. Guidance on the consent process, while lacking to some extent in the context of large-scale genomic studies, varies depending on jurisdiction, and standardizing consent processes internationally presents great challenges [4,5]. Having said this, clarifying data use groups that result in large part from the information having been provided at the time of consent is likely to provide greater understanding of downstream permissions and restrictions on the use of data generated for research studies. It may also be at this level that standardization of data use conditions can best be achieved. Standard data use conditions may also permit “bridging” consent between areas of research with different consent standards, such as disease-specific clinical research and population biobanking [6].

**Table 1:** Data use categories and requirements (Consent Codes): definition and abbreviation.

Consent Codes		
Name	Abbreviation	Description
Primary Categories (I^ry^)		
No restrictions	NRES	No restrictions on data use.
General research use and clinical care	GRU(CC)	For health/medical/biomedical purposes, including the study of population origins or ancestry.
Health/medical/biomedical research and clinical care	HMB(CC)	Use of the data is limited to health/medical/biomedical purposes; does not include the study of population origins or ancestry.
Disease-specific research and clinical care	DS-[XX](CC)	Use of the data must be related to [disease].
Population origins/ancestry research	POA	Use of the data is limited to the study of population origins or ancestry.
Secondary Categories (II^ry^) (can be one or more extra conditions, in addition to I^ry^ category)		
Oher research-specific restrictions	RS-[XX]	Use of the data is limited to studies of [research type] (e.g., pediatric research).
Research use only	RUO	Use of data is limited to research purposes (e.g., does not include its use in clinical care).
No “general methods” research	NMDS	Use of the data includes methods development research (e.g., development of software or algorithms) ONLY within the bounds of other data use limitations.
Genetic studies only	GSO	Use of the data is limited to genetic studies only (i.e., no “phenotype-only” research).
Requirements		
Not-for-profit use only	NPU	Use of the data is limited to not-for-profit organizations.
Publication required	PUB	Requestor agrees to make results of studies using the data available to the larger scientific community.
Collaboration required	COL-[XX]	Requestor must agree to collaboration with the primary study investigator(s).
Ethics approval required	IRB	Requestor must provide documentation of local IRB/REC approval.
Geographical restrictions	GS-[XX]	Use of the data is limited to within [geographic region].
Publication moratorium/embargo	MOR-[XX]	Requestor agrees not to publish results of studies until [date].
Time limits on use	TS-[XX]	Use of data is approved for [x months].
User-specific restrictions	US	Use of data is limited to use by approved users.
Project-specific restrictions	PS	Use of data is limited to use within an approved project.
Institution-specific restrictions	IS	Use of data is limited to use within an approved institution.

We divided data use conditions into nineteen data use Categories and Requirements. Requirements are conditions that would require some form of additional agreement on the part of the data provider or data user or additional criteria in order for the data to be reused. Data use Categories were further divided into primary (I^ry^) and secondary (II^ry^) Categories. All datasets should fall into only one primary Category, whereas a number of secondary Categories and Requirements may also apply to datasets. For the Beacon Project, assigning datasets to a primary Category has become mandatory so as to enable the inclusion of data that comes with some restrictions on secondary use.

We reused existing abbreviations from the National Institutes of Health (NIH) library of Standard Data Use Limitations (DULs) for the Categories and Requirements [7] to facilitate consistent interpretation of the appropriate secondary uses of the data and compatibility between different datasets. However, the definitions of several of these Categories have been expanded with respect to the use of data in research and in clinical care (see discussion below for GRU, HMB, and DS-[XX]). This is shown by the abbreviation (CC), for “clinical care.”

Here, we discuss the main issues that were raised by the categorization of data use conditions and our rationale for the choices made.

### No restrictions

A “no restrictions” data use Category was included primarily for datasets for which consent to an “open” dataset to be placed in an unrestricted online access repository (e.g., HapMap, 1000 Genomes Project) has been provided. It could also be used for datasets for which consent to data sharing is usually not required (e.g., summary statistics).

### Research and clinical care

A major issue we sought to address is that of dataset generation and use in both research and clinical contexts. An example of this is sharing either anonymized or coded patient information to find similar cases elsewhere among colleagues around the world. A goal of the Global Alliance Matchmaker Exchange (MME) project is to facilitate the use of coded patient information to enable discovery and, ultimately, clinical care.

In general terms, the need for consent to data sharing for either clinical care or research is usually dependent on the context in which data are collected and/or produced and shared, and on the balance between potential benefits and the probability of occurrence and seriousness of potential harm introduced by sharing possibly re-identifying information. Sharing within professional clinical networks is very important and often necessary for providing the best clinical care for patients. Nevertheless, broader accessibility and use of clinical datasets is potentially of great value to both scientific research and the clinical care it supports. As we foresee more clinical datasets being used in research in the future, we sought to identify any data use conditions that could specifically arise when seeking consent to data sharing for research purposes in a clinical setting, and we consulted researchers with experience in a clinical setting to this end. None were identified, which is, perhaps, not surprising, as many Database of Genotypes and Phenotypes (dbGaP) datasets, upon which much of the documentation we used was based, while considered typical research datasets, were partially generated in the clinic and include, for example, patient information about disease status.

Furthermore, when it is based on current standards of clinical practice, the sharing of clinical genomic datasets in support of clinical care may sometimes rely on presumed (or implied) consent—for example, for the use of data derived from residual medical samples [8]. We also sought to clarify if additional data use conditions would result from relying on presumed consent to clinical care. While we considered including a “clinical use only” category, this ultimately seemed unsatisfactory, as it would result in a silo of data with unclear permissions, whereas sufficient protection of clinical data could be achieved with the “health/medical/biomedical research and clinical care” or “disease-specific research and clinical care” data use Categories, along with the “collaboration required” data use Requirement for more complex data sharing agreements.

Conversely, it was important to consider the use of research datasets in the clinic. We expanded standard “research use” categories to “research and clinical care” in general terms with the understanding that, due to data quality and the likely consent situation, most of the time, research data would not be used directly in clinical decision-making (by researchers, clinicians, or others) or to provide research participants with their genetic information. If an obligation to share genetic data with research participants exists—and this may increasingly appear—it could be included under the “collaboration required” data use Requirement, which we imagine would be used to regroup different kinds of collaborative requirements (e.g., return of results, co-publication), though some of them might not be based on consent. As a separate matter, data quality, which may be of clinical grade, should be conveyed to data users. Defining data use Categories as for use in research and clinical care is, therefore, meant to reflect the pervasive contribution of scientific and medical research to inform clinical care, albeit in a somewhat indirect fashion for now. Along with this, a “research use only” II^ry^ data use Category was included, as a safeguard if participants were given assurances about limitations as to research use of data. Finally, it is important to address concerns about inclusion of research datasets in databases that may affect commercial services available to participants [9] through legal and other protections.

MME provides a good example of the overlap between research and clinical care uses of data. Patient data (typically phenotype and candidate genes from genomic analyses), which may have been collected in either a research or clinical care setting, are submitted during a broad query of networked databases. The goal is to find "matches" that may build evidence for implicating a new genetic cause of a disease phenotype. In this setting, both clinical care and research are facilitated, and the need for consent, either for clinical care or for research, is dependent on the detail and type of data shared [10].

As we enter the era of "learning healthcare," where every clinical encounter contributes to research, and research is being applied in real time to clinical care (e.g., for clinical sequencing or clinical trial entry in cancer treatment), we expect to see an ever greater blurring of research and clinical care boundaries.

### Methods research

Another important question was how to represent data use for statistical methods development research. Statistical methods development research is an essential domain within research—indeed, it is inextricable from research—and, similarly to disease-focused research, relies on access to data. Of 945 applications for access to the Genetic Association Information Network (GAIN) data received through 2011, 26% were for methods development [11]. As methods typically lead to applications that may be broader than the scope of the research for which they have been developed, methods development research raised questions with respect to the scope of patient and research participant consent, as it is often overlooked in descriptions of research projects. On the other hand, such research usually presents considerably less risk for data subjects, as datasets are not used to answer a specific research question, such as “What does this individual’s genetic information signify in this context?” Here, we can draw a comparison with the distinction between quality assurance and research. Quality assurance presents fewer ethical or legal risks than research with regard to the use of human biological materials and data, which are used to test a wider apparatus rather than for their research analysis [12]. The II^ry^ Category “no general methods research” was included for situations in which it would not seem acceptable to allow methods development research generally, and it would be limited by other data use conditions of the dataset. Our general assumption, therefore, is that statistical methods development research is permitted unless denoted by this II^ry^ Category (“no general methods research”).

### Publication moratorium/embargo

While conditions of publication on research results, such as a publication moratorium (or publication embargo), are not based on consent, we included a data use Requirement for these conditions, as they exist within the research community and ought to be clearly communicated to data users.

### Use of ontologies

The growing number of genomic datasets to be shared and reused is requiring machine-readability of data use conditions, including descriptions of types of data, details of sequencing protocols, variant types, and detailed patient data, along with our consent-based data use conditions. A growing number of ontologies are available to describe these and make them computable. For example, the Sequence Ontology [13,14] is a set of terms and relationships used to describe the features and attributes of biological sequence; for example, binding_site or exon. Similarly, ontologies are available to describe diseases (e.g., Disease Ontology [15] and Orphanet [16]) and phenotypes (Human Phenotype Ontology [HPO] [17]) and can be used for research restrictions based on disease area. In particular, the HPO has been widely adopted by many Global Alliance member institutions for detailed patient-level data.

In order to facilitate the application of data use conditions in software and tool development, we are considering integration of this proposed framework with one or more metadata and digital object models. We mention (i) the recently developed FAIR data principles (Findable, Accessible, Interoperable, Reusable; Mons et al. manuscript in preparation) [18]; (ii) ISA (Investigation, Study, Assay), a model for capturing research information originally for submission to EBI repositories [19]; (iii) research objects, a mechanism to bundle and annotate any kind of artefact related to an experiment [20]; and (iv) nanopublication, a newly developed mechanism to publish the smallest possible statements, such as a protein–protein interaction or a gene–disease association [21,22]. Although these models are usually very generic, they can relatively easily be extended, for instance, by using the aforementioned ontologies. The categories that we present here provide a basis for extensions that capture these data use conditions, thereby attaching them to a specific dataset. In this way, tools can be built that enable users to make more efficient use of the data use conditions as captured in the metadata of the digital object. This may include, for example, automated checks for compliance to conditions, condition-dependent search and navigation through large data collections, and facilitating automated negotiation of conditions in any type of data exchange.

## Conclusion

Our aim has been to develop a systematic set of Consent Codes for sites hosting data and for those preparing data for submission to the public archives to use in metadata descriptions of data. Their use should avoid introducing unnecessary new restrictions on data use while at the same time facilitating research with the greatest amount of suitable data available, based upon consents and the corresponding Consent Codes. Discussing the codes with ethics and governance committees (e.g., Institutional Review Boards [IRBs], Research Ethics Committees [RECs]) involved in research preparations, especially in reviewing consent protocols, will be important to facilitate their optimal use.

In the Beacon Project, the codes can be used to systematically disclose restrictions on use and to match the visibility of datasets shared through Beacon with investigators’ planned use of them. For clinical exchange services and in data discovery, the codes would help users identify host sites with data that would be suitable for their planned activities. We expect the tables to evolve over time as data sharing policy evolves and as more clinical and healthcare sites start engaging in real data exchange. The process of integrating the Consent Codes with computational tools may reveal outstanding uncertainties, which we will aim to resolve as they arise.

Current policy developments in the United States are relevant to this discussion. First, proposed changes to the Common Rule may encourage seeking broader consent to research purposes [23]. Second, from January 2015, many NIH-funded studies must share genomic data to the greatest extent possible, accompanied by the expectation that participants in these studies should provide consent to such sharing of genomic data [24].

## Methods

### Data use conditions

Our study of data use conditions included: (1) a review of guidance from the NIH; (2) a survey of data use conditions for data accessed within a large research institute, the Broad Institute of MIT and Harvard, and for data held within the EGA [25] of the EMBL-EBI and CRG; and (3) consultation with the international scientific community via the Global Alliance Data Working Group and with the MME project. Taken together, these provided us with a broad overview and evidence from a large number of datasets that are representative of the field.

The NIH expects that any limitations on the research use of the data, as expressed in the informed consent documents, should be clearly delineated in the institution’s assurance of the data submission to the dbGaP of the National Center for Biotechnology Information (NCBI) [26], known as the Institutional Certification [27]. For example, in some studies in dbGaP, a subset of research participants in a study may have provided informed consent that their data could only be used by not-for-profit companies that have no interest in commercializing a product as a result of the secondary data use. In an effort to provide greater consistency among the commonly used groups of limitations, the NIH developed a library of DULs [7]. In this case, the NIH standard DUL for this subgroup of participants would be “not-for-profit use only,” to indicate that, based on the original informed consent of the study participants, the data can be used only by not-for-profit organizations. To access “controlled-access” dbGaP datasets, researchers must submit a data access request (DAR), which includes a proposed research use statement, as well as agreement to the terms of use as described in the Data Use Certification Agreement (DUC) and attestation to the elements in the Genomic Data User Code of Conduct. The DULs for each of the datasets being requested are stipulated in the request process and the final DAR application. The relevant Data Access Committee (DAC) [28] reviews the DARs, and its decisions are based primarily upon conformity of the proposed research, as described in the DAR, to the DULs specified by the submitting institution.

The EGA follows a similar procedure for access to the controlled-access datasets it stores: Researchers requesting data apply to a given DAC in order to be granted access to the controlled data [29]. Each DAC reviews whether the applicant’s research purpose fits with the provided controlled Data Access Agreement (DAA) with the data donors. A difference with dbGaP is that the DAC process is not centralized, and each DAC has its own policy for gathering the information relating to application acceptance or rejection.

We also incorporated the findings of a research study that analyzed data use limitations in DUCs and other access agreements for datasets used at the Broad Institute of MIT and Harvard. This identified additional data use conditions as well as provided an estimate of their preponderance. Finally, we also incorporated knowledge of additional data use conditions from the experience of the EGA and of the members of the Global Alliance Data Working Group [30]. As the material we drew upon stemmed largely from experience with research datasets, we also sought a clinical genetics perspective from leaders of the MME project [31], a collaborative initiative to share data to enable rare disease gene discovery and supported by the Global Alliance, the International Rare Disease Research Consortium (IRDiRC) [32], ClinGen [33], and the individual MME services. We note that the MME and Beacon projects represent distinct approaches to data sharing. The MME provides access to extremely detailed patient data (e.g., matching case-level detail and data owner) and requires logging into an MME service and depositing case-level data such as phenotype and gene/genotype. The structure of the Beacon project allows for very easy access to data, but the data shared are less detailed (currently only the existence of a variant).

### Data use categories and requirements

We identified 19 categories of data use conditions and clarified their descriptions. These were then classified into “primary categories” or “secondary categories” and as “requirements,” based on their occurrence and on the resulting criteria for data access.

For validation purposes and to ensure as much compatibility as possible, we compared our classification to how the NIH ascribed dbGaP Consent Groups based on the NIH DULs, and to the DAA categorization performed by the EGA on the DAAs related to the studies with data stored in it. We included the abbreviations adopted by the NIH for overlapping data use conditions (e.g., GRU, HMB).
